# Identifying targets for antibiotic stewardship interventions through analysis of the antibiotic prescribing process in hospitals - a multicentre observational cohort study

**DOI:** 10.1186/s13756-020-00749-y

**Published:** 2020-07-21

**Authors:** Jannicke Slettli Wathne, Brita Skodvin, Esmita Charani, Stig Harthug, Hege Salvesen Blix, Roy M. Nilsen, Lars Kåre Selland Kleppe, Marta Vukovic, Ingrid Smith

**Affiliations:** 1 Department of Clinical Science, University of Bergen, Jonas Lies vei 87, 5021 Bergen, Norway; 2 Norwegian Advisory Unit for Antibiotic Use in Hospitals, Department of Research and Development, Haukeland University Hospital, Jonas Lies vei 65, 5021 Bergen, Norway; 3 Department of Quality and Development, Hospital Pharmacies Enterprise in Western Norway, Møllendalsbakken 9, 5021 Bergen, Norway; 4 NHIR Health Protection Research Unit in Healthcare Associated Infections and Antimicrobial Resistance, Imperial College London, Hammersmith Hospital Campus, Du Cane Road, London, W12 0NN UK; 5 Department of Drug Statistics, Norwegian Institute of Public Health, Marcus Thranes gate 6, 0473 Oslo, Norway; 6 School of Pharmacy, University of Oslo, Sem Sælandsvei 3, 0371 Oslo, Norway; 7 Western Norway University of Applied Sciences, Inndalsveien 28, 5063 Bergen, Norway; 8 Department of Infectious Diseases and Unit for Infection Prevention and Control, Department of Research and Education, Stavanger University Hospital, Armauer Hansens vei 20, 4011 Stavanger, Norway; 9 Department of Pharmaceutical Services, Oslo Hospital Pharmacy, Kirkeveien 166, 0450 Oslo, Norway; 10 Innovation, Access and Use, Department of Essential Medicines and Health Products, World Health Organization (WHO), Avenue Appia 20, 1211 Geneva 27, Switzerland

**Keywords:** Antimicrobial, Stewardship, Antibiotic, Prescribing, Process, AWaRe, Guideline, Hospital, Target, Intervention

## Abstract

**Background:**

In order to change antibiotic prescribing behaviour, we need to understand the prescribing process. The aim of this study was to identify targets for antibiotic stewardship interventions in hospitals through analysis of the antibiotic prescribing process from admission to discharge across five groups of infectious diseases.

**Methods:**

We conducted a multi-centre, observational cohort study, including patients with lower respiratory tract infections, exacerbation of chronic obstructive pulmonary disease, skin- and soft tissue infections, urinary tract infections or sepsis, admitted to wards of infectious diseases, pulmonary medicine and gastroenterology at three teaching hospitals in Western Norway. Data was collected over a 5-month period and included antibiotics prescribed and administered during admission, antibiotics prescribed at discharge, length of antibiotic therapy, indication for treatment and discharge diagnoses, estimated glomerular filtration rate (eGFR) on admission, antibiotic allergies, place of initiation of therapy, admittance from an institution, patient demographics and outcome data. Primary outcome measure was antibiotic use throughout the hospital stay, analysed by WHO AWaRe-categories and adherence to guideline. Secondary outcome measures were a) antibiotic prescribing patterns by groups of diagnoses, which were analysed using descriptive statistics and b) non-adherence to the national antibiotic guidelines, analysed using multivariate logistic regression.

**Results:**

Through analysis of 1235 patient admissions, we identified five key targets for antibiotic stewardship interventions in our population of hospital inpatients; 1) adherence to guideline on initiation of treatment, as this increases the use of WHO Access-group antibiotics, 2) antibiotic prescribing in the emergency room (ER), as 83.6% of antibiotic therapy was initiated there, 3) understanding prescribing for patients admitted from other institutions, as this was significantly associated with non-adherence to guideline (OR = 1.44 95% CI 1.04, 2.00), 4) understanding cultural and contextual drives of antibiotic prescribing, as non-adherent prescribing differed significantly between the sites of initiation of therapy (between hospitals and ER versus ward) and 5) length of therapy, as days of antibiotic therapy was similar across a wide range of diagnoses and with prolonged therapy after discharge.

**Conclusions:**

Analysing the process of antibiotic prescribing in hospitals with patient-level data identified important targets for antibiotic stewardship interventions in hospitals.

## Background

Suboptimal use of antibiotics is a key driver of antibiotic resistance [1]. In order to improve the antibiotic prescribing process, we need to understand it. Historically, antibiotic sales statistics have been easy to collect, and are therefore widely used as a proxy indicator to monitor antibiotic prescribing [2–5]. Although analyses of antibiotic sales data are useful at an aggregated level, they do not specify patient level use or outcomes. Whilst providing a baseline, such data cannot be used to assess the appropriateness of antibiotic prescribing, limiting opportunities for optimising antibiotic stewardship interventions. Accurate, patient level assessment of antibiotic prescribing is an essential step in optimising antibiotic use. Audit and prevalence studies with manual data collection are time-consuming, but often necessary to retrieve this information. Many hospitals still lack electronic medical records that allow automated extraction of antibiotic prescription data with accompanying indications for treatment [6–8]. The introduction of WHO Access, Watch and Reserve (AWaRe) categories have provided a framework for analysing antibiotic consumption, focusing on limiting unnecessary use of watch and reserve antibiotics [9, 10]. We present the findings of an observational multicentre cohort study aiming to identify targets for antibiotic stewardship interventions by analysing the antibiotic prescribing process from admission to discharge for individual patients.

## Methods

### Study design and setting

This was an observational, multicentre cohort study across the wards of infectious diseases, pulmonary medicine and gastroenterology at three teaching hospitals in Western Norway [11]. The largest hospitals (denoted A and B hereafter) are emergency care, university hospitals with 1100 and 600 beds, respectively, covering most specialities, except transplant surgery. Hospital C is an emergency care, teaching hospital with 160 beds, which is in close collaboration with Hospital A.

### Data collection

The cohort included patients recruited to an antibiotic stewardship intervention study and consisted of adult patients discharged from study wards between the 10th of February and the 11th of July 2014 with a hospital stay ≥24 h and ≤ 21 days, receiving antibiotics during admission for an indication within guideline recommendations [11, 12]. If a patient was readmitted during the study period, only the first stay was included. Patients with the following indications were included in the analysis: ^1)^ lower respiratory tract infections (LRTI) ^2)^ exacerbation of Chronic Obstructive Pulmonary Disease (COPD ex) ^3)^ urinary tract infections (UTI) ^4)^ skin- and soft tissue infections (SSTI) and ^5)^ sepsis. Patients were excluded if: a) they were admitted to intervention wards in the post-intervention period; and b) comorbidity and patient outcome data were missing.

Data were collected manually from electronic medical records, including admission notes from the emergency room, medical charts, physicians’ clinical notes, discharge letters and laboratory test results. Data included patient demographics, indication for antibiotic treatment, antibiotic use throughout the hospital stay, discharge diagnoses, estimated glomerular filtration rate (eGFR) on admission, length of stay, 30-day readmission, in-hospital and 30-day mortality, comorbidity and admittance from institution. Coded data on discharge diagnoses were retrieved from the hospital administrative system.

### Outcomes

The primary outcome measure was antibiotic regimens used throughout the hospital stay, grouped by AWaRe-categories and guidelines adherence on initiation of treatment and analysed at initiation of treatment, after first modification of regimen, and at discharge. Secondary outcome measures were antibiotic prescribing patterns by groups of diagnoses and non-adherence to the national antibiotic guidelines, analysed as association with study variables.

### Patient characteristics and diagnoses

To assess comorbidity, the Charlson Comorbidity Index (CCI) was calculated based on ICD-10 diagnoses at discharge [13, 14]. CCI was categorised as CCI equal to 0, 1, 2, 3, 4 or > 4, with zero being no registered comorbidity and > 4 substantial comorbidity.

The initial working diagnosis, documented in the electronic medical record for prescribed antibiotics, was used as the principal indication. Patients and treatment regimens were grouped by indications according to Supplement 1, Table 1. For patients having several diagnoses, all diagnoses were documented and a variable indicating multiple working diagnosis was created. Co-author BS (Infectious diseases (ID)-physician) assessed patients with multiple working diagnoses and assigned a primary indication for treatment based on the expectation that the treating physicians were likely to choose antibiotic treatment covering the most severe working diagnosis. Accuracy of diagnoses was defined as the percentage of patients for whom the initial indication for antibiotic treatment matched the discharge diagnosis (group level), defined as the infectious disease diagnosis coded or written in free text in the discharge letter.

### Antibiotic prescribing

Antibiotic regimens could include single or multiple antibiotics. Initially prescribed antibiotic regimens were assessed for adherence according to the Norwegian national antibiotic guidelines, as all hospitals included the national guidelines in their local antibiotic policy. Only first-choice empirical regimen for a given indication was regarded adherent. Assessment of adherence was performed using automated syntax in SPSS for Windows (IBM SPSS Statistics, version 24, USA). Indication for treatment was combined with prescribed active substance(s) to generate the adherence variable and adherence was thereafter adjusted manually for patients with kidney failure or antibiotic allergies.

Anti-infectives for systemic use (ATC-group J01), metronidazole tablets (ATC code P01AB01) and vancomycin tablets (A07AA09) were defined as antibiotics in this study. The prescribed antibiotic regimens were assigned to WHO AWaRe categories [9, 15]. For overview of AWaRe categories and included antibiotics, see Supplement 1, Table 2. Antibiotics belonging only to the “key access” category were included in the “access” category, while antibiotics belonging to “access-watch” and “watch” were included in the watch category. Since the use of antibiotics in the “reserve” category was minimal, the groups of “watch” and “reserve” were combined for analysis. Several antibiotics frequently used in Norway are not included in WHO AWaRe categories. To be able to include these patients in analysis, a modified version of AWaRe categories was prepared (Supplement 1, Table 2). Of the antibiotics not included in the original AWaRe categories, mecillinam, pivmecillinam, metenamin and tobramycin were added to the “access” category and cefuroxime was added to the “watch” category. If an antibiotic regimen contained both access and watch/reserve-group antibiotics, the regimen was classified as watch/reserve.

### Modification of antibiotic therapy

Modifications that prescribing physicians made to the first antibiotic regimen were defined in four categories: escalation, de-escalation, change within same level or unchanged. Day 1 was the day antibiotic therapy was initiated. Patients with regimens in the unchanged category were not included in analysis of time to change. Definitions of modifications are given in Table 1. Assessment of antimicrobial spectrum and categorisation of change were performed and checked by ID-physicians (authors BS and IS, respectively). Examples are given in Supplement 1, Table 3.

**Table 1:** Modifications of antibiotic regimens

**Process measures**	**Definition**
**Modification of therapy**	
Escalation	Change from oral to intravenous (i.v.) antibiotic treatment within the same antibacterial spectrum, change to more broad-spectrum treatment, adding an antibiotic to a combination.
De-escalation	Change from i.v. to oral antibiotic treatment within the same antibacterial spectrum or change to more narrow-spectrum treatment.
Change same level	Change to a regimen within the same antibacterial spectrum and form of administration (i.v./oral).
Unchanged	Regimens where first change of therapy was discontinuation of antibiotics, either during admission or after discharge.
**Time to first modification of AB regimen**	Time to first escalation/de-escalation/change within same antibacterial spectrum and dosage form (change of active substance(s), i.v. to oral switch, stopping or adding an antibiotic).
**Number of treatment regimens**	The number of treatment regimens from initiation of treatment until antibiotics prescribed at discharge
**Day of oral antibiotics**	The first day that one or more oral antibiotics were given.

### Duration of antibiotic therapy

Duration of antibiotic therapy was measured in days from the first to the last day of therapy and reported as: 1) mean total days of treatment, including prescribed treatment after discharge, 2) mean days of in-hospital antibiotic therapy and 3) mean days of therapy after discharge. When antibiotic treatment continued after discharge, the day of discharge was counted as in-hospital therapy. Information about antibiotic therapy after discharge was retrieved from the discharge letter and also reported as percentage of patients where post-discharge antibiotics were described.

### Data analysis

Descriptive statistics were applied to describe the prescription patterns. To examine which factors were associated with non-adherence, we used univariate and multivariate logistic regression. A targeted selection of factors were evaluated for the multivariate logistic regression model: place of antibiotic therapy initiation, indication for treatment, hospital site, admission from institution, accuracy between indication for treatment and discharge infection diagnosis, sex, age group, comorbidity measured by CCI, multiple working diagnoses, antibiotic allergies and eGFR. Variables that in univariate analysis had a *p*-value of less than 0.2 were included in the final model. Only the first four variables were associated with non-adherence in univariate analysis and included in the final multivariate model. *P*-values below 0.05 were considered statistically significant for all analysis. Stata SE version 15 (Stata Statistical Software, College Station, TX, USA) was used for all statistical analysis, while SPSS for Windows (IBM SPSS Statistics, version 24, USA) was used for assessment of adherence.

## Results

During the study period, 1544 patients with available comorbidity and outcome data met the inclusion criteria. Of these patients, 309 were admitted in the post-intervention period at intervention wards and was therefore excluded, leaving 1235 unique patients included in analysis for this study.

### Diagnoses and patient characteristics

The characteristics of the patients are given in Table 2. The most frequent diagnosis was LRTI (33.4%), followed by COPD exacerbations (22.7%), sepsis (20.1%), SSTI (12.2%) and UTI (11.7%) (not shown in tables). In the group of patients with SSTI, 6.0% of patients were admitted from an institution, compared to 20.7% for patients with UTI. When investigating accuracy between the groups of indications for empirical antibiotic treatment and discharge infection diagnoses, there was substantial variation with a range from 41.5% accuracy for patients initially diagnosed with sepsis, to 95.3% for patients diagnosed with SSTI.

**Table 2:** Patient characteristics

	**LRTI (*n*** **= 412) n (%)**	**COPD ex (*n*** **= 280) n (%)**	**Sepsis (*n*** **= 248) n (%)**	**SSTI (*n*** **= 150) n (%)**	**UTI (*n*** **= 145) n (%)**	**Total (*N*** **= 1235) n (%)**
**Sex**						
Male	196 (47.6)	149 (53.2)	148 (59.7)	101 (67.3)	62 (42.8)	656 (53.1)
Female	216 (52.4)	131 (46.8)	100 (40.3)	49 (32.7)	83 (57.2)	579 (46.9)
**Age**						
< = 45	43 (10.4)	2 (0.7)	51 (20.6)	52 (34.7)	16 (11.0)	164 (13.3)
46–65	88 (23.4)	70 (25.0)	50 (20.2)	48 (32.0)	21 (14.5)	277 (22.4)
66–85	192 (46.6)	179 (63.9)	106 (42.7)	37 (24.7)	71 (49.0)	585 (47.4)
> 85	89 (21.6)	29 (10.4)	41 (16.4)	13 (8.7)	37 (25.5)	209 (16.9)
**Charlson Comorbidity Index**						
CCI = 0	163 (39.6)	8 (2.9)	111 (44.8)	108 (72.0)	72 (49.7)	462 (37.4)
CCI = 1	109 (26.5)	178 (63.6)	73 (29.4)	23 (15.3)	37 (25.5)	420 (34.0)
CCI = 2	58 (14.1)	47 (16.8)	41 (16.5)	9 (6.0)	21 (14.5)	176 (14.3)
CCI = 3	32 (7.8)	24 (8.6)	9 (3.6)	5 (3.3)	9 (6.2)	79 (6.4)
CCI = 4	14 (3.4)	18 (6.4)	6 (2.4)	3 (2.0)	1 (0.7)	42 (3.4)
CCI > 4	36 (8.7)	5 (1.8)	8 (3.2)	2 (1.3)	5 (3.5)	56 (4.5)
**Admitted from institution**						
No	341 (82.8)	255 (91.1)	203 (81.9)	141 (94.0)	115 (79.3)	1055 (85.4)
Yes	71 (17.2)	25 (8.9)	45 (18.1)	9 (6.0)	30 (20.7)	180 (14.6)
**AB allergies**						
Yes	43 (10.5)	38 (13.6)	19 (7.7)	13 (8.7)	9 (6.2)	122 (9.9)
No	367 (89.3) 1 missing	242 (86.4)	229 (92.3)	137 (91.3)	136 (93.8)	1111 (90.0) 1 missing
**eGFR on admission**						
> 50	308 (74.8)	230 (82.1)	187 (75.4)	129 (86.0)	101 (69.7)	955 (77.3)
10–50	103 (25.0)	49 (17.5)	59 (23.8)	21 (14.0)	43 (29.7)	275 (22.3)
< 10	1 (0.24)	0 (0.0)	2 (0.8)	0 (0.0)	1 (0.7)	4 (0.32)
Dialysis	0 (0.0)	1 (0.4)	0 (0.0)	0 (0.0)	0 (0.0)	1 (0.08)
**30-day mortality**	55 (13.4)	19 (6.8)	22 (8.9)	2 (1.3)	6 (4.1)	104 (8.4)
**30-day readmission**	78 (18.9)	76 (27.1)	39 (15.7)	26 (17.3)	37 (25.5)	256 (20.7)
**Mean LOS (95% CI)**	7.3 (6.8, 7.7)	6.8 (6.3, 7.2)	7.1 (6.6, 7.6)	6.3 (5.6, 7.0)	7.0 (6.3, 7.7)	7.0 (6.7, 7.2)

### Empirical antibiotic prescribing

**Fig. 1:**
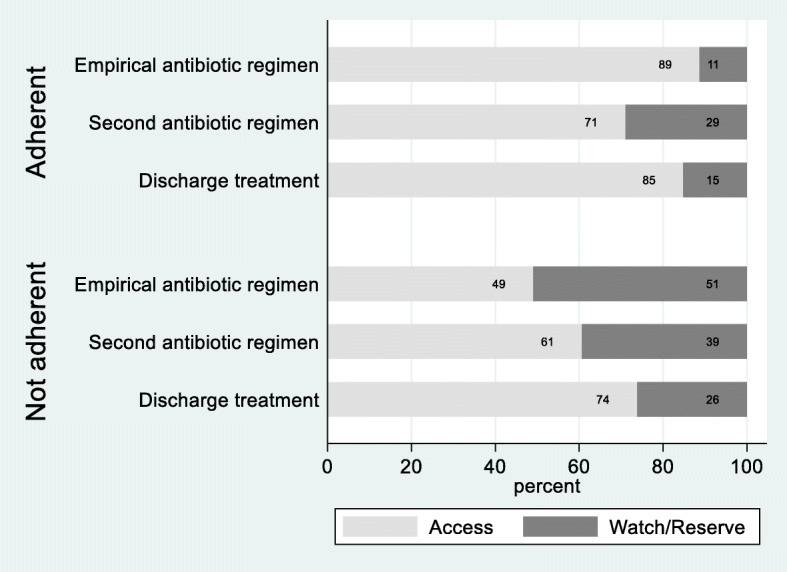
Antibiotic regimens prescribed from admission to discharge, by AWaRe categories and adherence to guideline on initiation of therapy

Prescribed antibiotic regimens were adherent to guidelines for 63% of patients (Table 3). Antibiotics belonging to the WHO AWaRe “Access” category were prescribed as initial regimen for 74% of patients in total, while the remaining 26.0% of antibiotic regimens were from the “Watch/Reserve” category. Where initial antibiotic regimens were adherent to guidelines, 89% of regimens were in the WHO AWaRe access category (Fig. 1). Second regimens included more antibiotics from the watch/reserve categories and 71% of regimens were now in the access category. At discharge, 85% of regimens from the adherent group were in the access category. Where initial antibiotic regimens were non-adherent to guidelines, 49% of the regimens were in the access category. This increased to 61% for the second regimen and then again to 74% of regimens being the access category at discharge.

**Table 3:** Antibiotic (AB) prescribing in hospitals – process measures

	**LRTI (*n*** **= 412) n (%)**	**COPD ex (*n*** **= 280) n (%)**	**Sepsis (*n*** **= 248) n (%)**	**SSTI (*n*** **= 150) n (%)**	**UTI (*n*** **= 145) n (%)**	**Total (*N*** **= 1235) n (%)**
**AB initiated**						
Emergency room	320 (77.7)	244 (87.1)	240 (96.8)	135 (90.0)	94 (64.8)	1033 (83.6)
Ward	92 (22.3)	36 (12.9)	8 (3.2)	15 (10.0)	51 (35.2)	202 (16.4)
**Adherence to guideline**						
Yes	280 (68.0)	177 (63.2)	151 (60.9)	90 (60.0)	80 (55.2)	778 (63.0)
No	132 (32.0)	103 (36.8)	97 (39.1)	60 (40.0)	65 (44.8)	457 (37.0)
**Accuracy between indication for AB-treatment and discharge infection diagnoses**^a^						
Yes	331 (80.3)	255 (91.1)	103 (41.5)	143 (95.3)	122 (84.1)	954 (77.3)
No	81 (19.7)	25 (8.9)	145 (58.5)	7 (4.7)	23 (15.9)	281 (22.8)
**Empirical AB regimen was**						
Changed during admission	232 (56.3)	167 (59.6)	205 (82.7)	82 (54.7)	73 (50.3)	759 (61.4)
Changed at discharge	99 (24.0)	57 (20.4)	22 (8.9)	57 (38.0)	19 (13.1)	254 (20.6)
Continued at discharge	32 (7.8)	29 (10.4)	2 (0.8)	5 (3.3)	34 (23.5)	102 (8.3)
Stopped	49 (11.9)	27 (9.6)	19 (7.7)	6 (4.0)	19 (13.1)	120 (9.7)
**Empirical AB regimen was**						
De-escalated	227 (55.1)	176 (62.9)	142 (57.3)	95 (63.3)	56 (38.6)	696 (56.4)
Escalated	84 (20.4)	45 (16.1)	43 (17.3)	29 (19.3)	24 (16.5)	225 (18.2)
Changed-equal spectrum	19 (4.6)	3 (1.1)	41 (16.5)	15 (10.0)	12 (8.3)	90 (7.3)
Unchanged^b^	82 (19.9)	56 (20.0)	22 (8.9)	11 (7.3)	53 (36.6)	224 (18.1)
**Time to change of first AB regimen (*n*** **= 1011)**						
Mean (95% CI)^c^	4.0 (3.7, 4.2)	3.9 (3.7, 4.1)	3.0 (2.7, 3.3)	3.4 (3.1, 3.8)	3.5 (3.1, 3.9)	3.6 (3.5, 3.8)
**Number of treatment regimens through admission**						
1	41 (9.9)	33 (11.8)	5 (2.0)	6 (4.0)	34 (23.5)	119 (9.6)
2	245 (59.5)	187 (66.8)	86 (34.7)	77 (51.3)	75 (51.7)	670 (54.3)
3	92 (22.3)	43 (15.4)	122 (49.2)	44 (29.3)	29 (20.0)	330 (26.7)
> 3	34 (8.3)	17 (6.1)	35 (14.1)	23 (15.3)	7 (4.8)	116 (9.4)
**Oral AB given**						
Yes	326 (79.1)	243 (86.8)	204 (82.3)	136 (90.7)	135 (93.1)	1044 (84.5)
No	86 (20.9)	37 (13.2)	44 (17.7)	14 (9.3)	10 (6.9)	191 (15.5)
Mean first day (95% CI)	4.2 (3.9, 4.5)	3.6 (3.3, 3.8)	5.1 (4.6, 5.5)	4.8 (4.3, 5.3)	2.7 (2.3, 3.1)	4.1 (3.9, 4.3)
**First change of AB regimen**						
During admission	232 (56.3)	167 (59.6)	205 (82.7)	82 (54.7)	73 (50.3)	759 (61.5)
At discharge	99 (24.0)	57 (20.4)	22 (8.9)	57 (38.0)	19 (13.1)	254 (20.6)
Continued at discharge	32 (7.8)	29 (10.4)	2 (0.8)	5 (3.3)	34 (23.5)	102 (8.3)
Stopped	49 (11.9)	27 (9.6)	19 (7.7)	6 (4.0)	19 (13.1)	120 (9.7)
**Antibiotics prescribed at discharge**						
Yes	296 (71.8)	214 (76.4)	193 (77.8)	139 (92.7)	114 (78.6)	956 (77.4)
No	116 (28.2)	66 (23.6)	55 (22.2)	11 (7.3)	31 (21.4)	279 (22.6)
**Days of AB treatment**						
Mean (95% CI)^d^	10.2 (9.7, 10.6)	10.0 (9.6, 10.5)	11.5 (10.8, 12.2)	12.5 (11.6, 13.4)	9.3 (8.6, 10.1)	10.6 (10.3, 10.9)
In-hospital	6.3 (5.9, 6.7)	6.0 (5.7, 6.4)	6.6 (6.1, 7.1)	6.0 (5.3, 6.6)	5.3 (4.8, 5.8)	6.2 (5.9, 6.4)
After discharge^e^	5.5 (5.2, 5.8)	5.2 (4.9, 5.6)	6.3 (5.8, 6.8)	7.1 (6.4, 7.7)	4.9 (4.5, 5.3)	5.8 (5.6, 6.0)

^a^ Measured as match between initial grouped indication for treatment and grouped discharge diagnosis

^b^ "Unchanged" includes patients where discontinuation of antibiotics was the only change

^c^ Does not include patients who did not change initial antibiotic regimen (stop was only change)

^d^ Does not include 40 patients where lenght of prescription treatment after discharge not stated in the discharge letter

^e^ Does not include 40 patients where length of prescription treatment was not stated in the discharge letter and 5 patients where length of prescriptions treatment was longer than 30 days

The majority (83.6%) of antibiotic prescriptions were initiated in the emergency room, ranging from 64.8% of prescriptions for UTI to 96.8% for sepsis (Table 3). Initiating antibiotic therapy at the ward increased the likelihood for non-adherence to guidelines, compared to prescribing in the emergency room, with an odds-ratio (OR) of 1.7, 95% CI (1.24, 2.36) (Table 4). When compared to LRTI, all groups of diagnoses were associated with a higher likelihood of non-adherence, ranging from OR = 1.42, 95% CI (1.03, 1.98) for COPD ex to OR = 1.62, 95% CI (1.09, 2.41) for UTI (all *p* < 0.05). Being admitted to hospital B was associated with reduced OR of non-adherence compared to hospital A, with an OR = 0.63, 95% CI (0.46, 0.86), *p* = 0.004. Patients admitted from an institution had increased risk of receiving non-adherent antibiotic treatment, OR = 1.44, 95% CI (1.04, 2.00), *p* = 0.029. Other factors tested were not associated with prescriptions being non-adherent to guidelines.

**Table 4:** Factors associated with non-adherence to antibiotic guideline

	**Adherence (*n*** **= 778) n (%)**	**Non-adherence (*n* = 457) n (%)**	**Univariate analysis OR (95% CI)**	***p*-value**	**Adjusted analysis**^**a**^**OR (95% CI)**	***p*-value**
**AB initiated**						
Emergency room	670 (64.9)	363 (35.1)	1		1	
Ward	108 (53.5)	94 (46.5)	1.6 (1.18, 2.18)	**0.002**	1.7 (1.24, 2.36)	**0.001**
**Indication for treatment**						
LRTI	280 (68.0)	132 (32.0)	1		1	
COPD ex	177 (63.2)	103 (36.8)	1.23 (0.90, 1.70)	0.196	1.42 (1.03, 1.98)	**0.035**
Sepsis	151 (60.9)	97 (39.1)	1.36 (0.98, 1.89)	0.065	1.44 (1.02, 2.02)	**0.037**
SSTI	90 (60.0)	60 (40.0)	1.41 (0.96, 2.10)	0.079	1.56 (1.05, 2.31)	**0.028**
UTI	80 (55.2)	65 (44.8)	1.72 (1.17, 2.54)	**0.006**	1.62 (1.09, 2.41)	**0.017**
**Hospital**						
Hospital A	376 (60.8)	242 (39.2)	1		1	
Hospital B	203 (70.7)	84 (29.3)	0.64 (0.48, 0.87)	**0.004**	0.63 (0.46, 0.86)	**0.004**
Hospital C	199 (60.3)	131 (39.7)	1.02 (0.78, 1.34)	0.872	0.95 (0.71, 1.26)	0.712
**Admitted from institution**						
No	678 (64.3)	377 (35.7)	1			
Yes	100 (55.6)	80 (44.4)	1.44 (1.04, 1.98)	**0.026**	1.44 (1.04, 2.00)	**0.029**
**Accuracy between indication for AB-treatment and discharge infection diagnoses**^b^						
Yes	604 (63.3)	350 (36.7)	1		1	
No	174 (61.9)	107 (38.1)	1.06 (0.81, 1.40)	0.671	0.99 (0.72, 1.35)	0.936
**Sex**						
Male	411 (62.7)	245 (37.3)	1		1	
Female	367 (63.4)	212 (36.6)	0.97 (0.77. 1.22)	0.790	0.98 (0.77, 1.24)	0.857
**Age**						
< 45	106 (64.6)	58 (35.4)	1		1	
46–65	168 (60.7)	109 (39.3)	1.19 (0.79, 1.77)	0.405	1.26 (0.83, 1.91)	0.277
66–85	367 (62.7)	218 (37.3)	1.09 (0.76, 1.56)	0.656	1.08 (0.73, 1.60)	0.695
> 85	137 (65.6)	72 (34.4)	0.96 (0.63, 1.47)	0.854	0.87 (0.54, 1.37)	0.540
**Charlson Comorbidity Index**						
CCI = 0	304 (65.8)	158 (34.2)	1		1	
CCI = 1	265 (63.1)	155 (36.9)	1.13 (0.85, 1.48)	0.402	1.14 (0.83, 1.56)	0.421
CCI = 2	102 (57.9)	74 (42.1)	1.40 (0.98, 1.99)	0.066	1.35 (0.93, 1.97)	0.115
CCI = 3	48 (60.8)	31 (39.2)	1.24 (0.76, 2.10)	0.386	1.20 (0.72, 2.02)	0.482
CCI = 4	26 (61.9)	16 (38.1)	1.18 (0.62, 2.27)	0.611	1.18 (0.60, 2.33)	0.626
CCI > 4	33 (58.9)	23 (41.1)	1.34 (0.76, 2.36)	0.310	1.39 (0.77, 2.51)	0.279
**Number of working diagnoses**						
1	493 (64.6)	270 (35.4)	1		1	
2	238 (60.7)	154 (39.3)	1.18 (0.92, 1.52)	0.193	1.12 (0.87, 1.46)	0.381
3	47 (58.8)	33 (41.2)	1.28 (0.80, 2.05)	0.299	1.18 (0.73, 1.92)	0.491
**Antibiotic allergies**^**c**^						
No	708 (63.7)	403 (36.3)	1		1	
Yes	69 (56.6)	53 (43.4)	1.35 (0.92, 1.97)	0.120	1.40 (0.95, 2.06)	0.088
**eGFR**						
eGFR > 50	598 (62.6)	357 (37.4)	1		1	
eGFR < 50	180 (64.3)	100 (35.7)	0.93 (0.71, 1.23)	0.611	0.88 (0.66, 1.17)	0.378

^a^ All factors are adjusted for where AB was initiated, indication for AB treatment, hospital and admittance from institution

^b^ Measured as match between initial grouped indication for treatment and grouped discharge diagnosis

^c^1 data missing

### Modification of antibiotic therapy

The initial antibiotic regimen was modified during admission for 61.4% of the patients, and 20.6% of initial regimens was continued until discharge and then changed (Table 3). For the remaining patients, the initial antibiotic regimen was either stopped (9.7%) or continued after discharge (8.3%). This pattern varied between diagnoses. For patients with sepsis, 82.7% of initial antibiotic regimens were changed during admission, in contrast to 54.7 and 50.3% of regimens for SSTI and UTI patients, respectively.

De-escalation was the most frequent first modification of antibiotic regimens and in total, 56.4% of first modifications were de-escalations, across all diagnoses (Table 3). For patients whose therapy was modified, the mean day of change was 3.6 days with 95% CI (3.5, 3.8). The time from start of antibiotic therapy to first change varied from patients with sepsis where day 3.0 with 95% CI (2.7, 3.3) was the mean day of change to patients with LRTI where change occurred on day 4.0 with 95% CI (3.7, 4.2).

In total, 84.5% of patients received oral antibiotics during the course of treatment (Table 3). Time to oral treatment differed substantially between diagnoses, from 2.7 days, 95% CI (2.3, 3.1) for UTI’s to 5.1 days, 95% CI (4.6, 5.5) for sepsis.

### Duration of antibiotic therapy

The mean duration (in-house and post-discharge) of antibiotic therapy was 10.6 days, 95% CI (10.3, 10.9) (Table 3). Mean days of in-house and post-discharge therapy was similar across all diagnosis. Patients diagnosed with sepsis had the highest mean number of in-house antibiotic days at 6.6 days, 95% CI (6.1, 7.1), while patients with SSTI had the highest mean days of therapy after discharge and total days of antibiotics with 7.1, 95% CI (6.4, 7.7) and 12.5 days 95% CI (11.6, 13.4), respectively. After discharge, 77.4% of patients continued with antibiotic therapy.

## Discussion

This study has identified key gaps and potential targets in the antibiotic prescribing process in hospitals for antibiotic stewardship interventions (Table 5).

**Table 5:** Identified gaps and potential targets for antibiotic stewardship interventions

**Gaps identified**	**Potential targets**
Guideline adherence increased the use of narrow spectrum WHO Access group antibiotics in this study setting	Promoting adherence to guidelines when prescribing empirical antibiotic therapy
Antibiotic therapy was initiated in the emergency room for 83.6% of patients	Targeting antibiotic prescribing in the emergency room, focusing on first line clinical staff
Non-adherence to antibiotic guideline was associated with admittance from another institution	Understanding the drivers for non-adherence in patients admitted from institutions and focusing on antibiotic prescribing for this group of patients
Non-adherence to antibiotic guideline was associated with the place of initiation of therapy, both regarding hospital site and wards compared to emergency room	Understanding the cultural and contextual drivers for antibiotic prescribing across institutions and specialties
Mean length of antibiotic therapy was similar across very different groups of diagnosis.	Focusing on reducing the duration of antibiotic therapy safely, in accordance with emerging evidence on duration of antibiotic treatment
Antibiotics prescribed upon discharge contributed significantly to the total days of antibiotic therapy and the appropriateness of this practice is often not clear	

One of the main aims of antibiotic stewardship programs is to reduce unnecessary use of broad-spectrum antibiotics. We applied WHO AWaRe categories to describe the categories of antibiotics prescribed and found that when initial antibiotic treatment were according to Norwegian national guidelines, the majority of regimens (89%) consisted of only access group antibiotics. Non-adherent empirical regimens however, included several antibiotics from the watch/reserve category, but these regimens were often switched to regimens within the access categories upon first modification of treatment. At discharge, a greater number of regimens were from the access category, both suggestive of clinical microsystems that tried to adhere to guidelines and antibiotic stewardship principles, but also likely related to the restricted availability of oral broad-spectrum antibiotics in Norway.

In an American study from 2014, Braykov et al. ranked antibiotics in categories of narrow-spectrum, broad-spectum, extended spectrum and restricted antibiotics [16]. Although there are some differences between the studies regarding the categories used to classify antibiotics, the results show that the prescription pattern is very different between the hospitals in the two studies. While 74% of patients in our study initially received antibiotics belonging only to the access group, most patients (78%) had broad-spectrum and extended spectrum antibiotics prescribed as empirical therapy in the Braykov-study. This reflects the nature of the Norwegian national antibiotic guidelines, which mainly have antibiotics from the access group as first-line empirical treatment recommendations.

Initiating empirical antibiotic therapy is a crucial step in the treatment of infections and an important target for antibiotic stewardship interventions, as recently outlined by Tamma et al. in their paper describing the four moments of antibiotic decision making [17]. In our study, antibiotics were mainly prescribed in the emergency departments. The physicians responsible for prescribing are usually interns and residents and in Norwegian hospitals, junior doctors rely heavily on guidelines for antibiotic prescribing [18]. From a separate study by Skodvin et al., including patients from the same intervention study cohort, we also know that mean compliance with guidelines recommendations for microbiology testing practices was 89% [19]. Most patients (83.6%) started antibiotic treatment in the emergency departments and non-adherence to guidelines was higher when treatment was initiated at the wards, compared to the emergency departments (OR = 1.7, 95% CI (1.24–2.36), *p* = 0.001). Other studies report reluctance from other medical teams to change therapy further down the line and together this highlights the need to focus on first-line clinical staff when planning antibiotic stewardship interventions [20].

Non-adherence to guidelines was also associated with hospital site and whether patients were admitted from an institution or not. Patients admitted from nursing homes or other institutions may have had treatment prior to hospital admission. A combination of age, frailty, comorbidity and increased fear of resistant bacteria as cause of infection could have caused physicians to prescribe more broad-spectrum antibiotics for these patients. There is however, a need for a more thorough understanding of prescribing practices in this particular group of patients. Studies show that organisational culture influence antibiotic prescribing [20–22]. This could potentially explain why the odds ratio for non-adherent prescribing was significantly lower at hospital B than the two other included hospitals (OR = 0.63 95% CI (0.46, 0.86), *p* = 0.004). It also signals that a thorough understanding of organisational culture with barriers and facilitators for prudent antibiotic prescribing is an important part of planning for antibiotic stewardship interventions.

Empirical antibiotic regimens were usually modified during admission (61.5%) and oral antibiotics were prescribed for 84.5% of patients. Other studies looking at the process of antibiotic prescribing in hospitals have focused on review of empirical therapy in relation to patient outcome or effect of interventions on prescribing process measures [23–25]. Braykov et al. found that by the 5th day of therapy, 21,5% of empirical antibiotics were narrowed or discontinued, while Aillet et al. found that antibiotic review was performed in 69% of patients with bacteraemia [16, 24]. In comparison, although we did not measure all patients at one specific day, 74,5% of empirical antibiotics were de-escalated (56,4%) and stopped (18,1%) as first modification of therapy in our study. Modifications happened between day 3 and 4 when initiation of therapy was defined as day 1. This is in agreement with recommendations stating that review of therapy should take place 48–72 h after initiation of antibiotic therapy [26–28]. Upon discharge, 77.4% of patients continued antibiotic treatment and the mean length of post-discharge therapy was similar to the mean length of in-house treatment. This could mean either that most patients were not fully recovered upon discharge or that antibiotics were continued “just in case,” justifying an earlier discharge and giving the physician reassurance for the patients’ well-being. The lack of documentation regarding length of antibiotic therapy has been heavily debated and studies suggest shorter antibiotic courses are safe and effective for an increasing number of diagnoses [29–33]. In our study, there was a remarkable similarity in duration of antibiotic therapy between the various groups of diagnoses, both in-hospital and post-discharge. For all patients, the mean number of days of antibiotic therapy were 10.6 days and the range for the various groups were narrow (9.3 to 12.5 days) when post-discharge therapy was included. There is a need for more studies, informing policymakers and clinicians about the optimal duration of antibiotic therapy for individual diagnosis, both in-hospital and for post-discharge use.

This study has some limitations. When assessing adherence to guidelines on initiation of treatment, we used the indication for treatment stated in the electronic medical record, and this was usually a working diagnosis on admission. The diagnosis may change with more data and results available. To check whether this constituted a major issue for interpretation of data, we looked at the coherence between indication for treatment and the infection discharge diagnosis (if present) in the discharge letter. The group of diagnoses for which this might be an issue, is sepsis, where accuracy between indication for treatment and discharge diagnosis was low. During the study period, SIRS-criteria were used to screen patients for sepsis. SIRS identify more patients with suspected sepsis than the qSOFA score, which is currently in use. The low accuracy in this group could be due to lack of documentation of sepsis at discharge, with only the original focus of the infection often documented in discharge papers. It is possible that review of therapy took place without modifications to the patient’s antibiotic regimen. Such reviews were not identified during data collection and represents a limitation to this study. We also did not take dosing into consideration when assessing adherence to guidelines and modification of therapy. Appropriateness of antibiotic therapy was not evaluated after initial assessment of adherence to guidelines for empirical antibiotic treatment. It is therefore unknown whether escalation, de-escalation, stop or change was the best option for each individual patient. From the study by Skodvin et al., with patients derived from the same intervention study cohort, we do however know that only 18% of patients had applicable microbiology test results and for only half of these patients (9% of the total cohort), these findings were used to guide therapy [19].

The Nordic countries and the Netherlands are currently in a favourable position regarding antimicrobial resistance and are still able to utilize the most ecologically friendly antibiotics in empirical regimens. Exploring different ways of aggregating and analysing data to understand hospital antibiotic prescribing processes are however important in all countries and institutions, aiming to identify targets for stewardship interventions.

Future studies should include assessment of appropriateness of therapy throughout the hospital stay to have a more comprehensive review of prescribing quality at every step of the process. Identifying and studying contributions from other healthcare professionals, like nurses and pharmacist and the team effort in antibiotic stewardship would also be valuable. As patient involvement and empowerment is increasing, the contribution of patients in antibiotic stewardship in hospital settings should also be investigated. Such studies could contribute to the identification of more targets for antibiotic stewardship interventions.

## Conclusions

Analysis of patient level antibiotic prescribing data and the use of WHO AWaRe to categorise antibiotic regimens throughout the hospital stay, identified relevant targets for antibiotic stewardship interventions in our population of hospital inpatients. Identified targets included 1) adherence to guidelines 2) focus on prescribing physicians in the emergency room 3) understanding prescribing for patients admitted from an institution 4) organisational culture and 5) duration of antibiotic therapy.

## Supplementary information

**Additional file 1: Table 1.** Grouping of indication for treatment. **Table 2.** Overview of AWaRe categories with study modifications. **Table 3.** Evaluation of antimicrobial spectrum and categorization of change.

## Data Availability

The datasets generated and/or analysed during the current study are not publicly available in concordance with the approval from the Data Protection Officer (2013/9352), but are available from the corresponding author on reasonable request.

## Abbreviations

AB: Antibiotic
AWaRe: Access, Watch, Reserve
CCI: Charlson Comorbidity Index
COPD ex: Exacerbation of Chronic Obstructive Pulmonary Disease
eGFR: Estimated Glomerular Filtration Rate
UTI: Urinary Tract Infection
LOS: Length of stay
LRTI: Lower Respiratory Tract Infection
SIRS: Systemic Inflammatory Response Syndrome
SSTI: Skin- and Soft Tissue Infections
qSOFA: Quick Sequential [Sepsis-related] Organ Failure Assessment score
WHO: World Health Organization
