# Supplementary material for: Identifying targets for antibiotic stewardship interventions through analysis of the antibiotic prescribing process in hospitals - a multicentre observational cohort study
Source: Antimicrob Resist Infect Control. 2020 Jul 21;9:114. doi: 10.1186/s13756-020-00749-y (PMC7374853; doi:10.1186/s13756-020-00749-y)
Supplement: Supplementary file 1 — Additional file 1: Table 1. Grouping of indication for treatment. Table 2. Overview of AWaRe categories with study modifications. Table 3. Evaluation of antimicrobial spectrum and categorization of change. [file 13756_2020_749_MOESM1_ESM.docx]

SUPPLEMENT 1

**Table 1: Grouping of indication for treatment**

|  | **Indication for treatment*** |
| --- | --- |
| Lower respiratory tract infections (LRTI) | Community acquired pneumonia (normal and severe), healthcare associated pneumonia (normal and severe), unspecified lower respiratory tract infections, unknown – suspected pneumonia, aspiration pneumonia, atypical pneumonia, lung abscess, empyema. |
| Chronic obstructive pulmonary disease exacerbation (COPD ex) | Patients with COPD, presenting with LRTI (community and healthcare associated). |
| Sepsis | Suspected cases of sepsis originating from; lower respiratory tract, urinary tract, unknown focus, soft tissue, abdomen and catheter. |
| Skin and soft tissue infections (SSTI) | Erysipelas, cellulitis, abscess, other skin and soft tissue infections, mastitis, necrotising soft tissue infections, postoperative wound infection. |
| Urinary tract infections (UTI) | UTI – unspecified, pyelonephritis, lower UTI/cystitis, unknown-suspected UTI, catheter associated UTI. |

*Indications are given in decreasing order of frequency

**Table 2:**

**Overview of AWaRe categories with study modifications**

| **AWaRe**  **Category** | **AWaRe antibiotic** | **Active substances** | **Study antibiotics added to category** |
| --- | --- | --- | --- |
| **Access*** | Beta-lactam antibiotics | Amoxicillin, amoxicillin + clavulanic acid, ampicillin, benzathine benzylpenicillin, benzylpenicillin, cefalexin, cefazolin, cloxacillin, phenoxymetylpenicillin,  procain benzyl penicillin | Pivmecillinam  Mecillinam  Cefalotin  Dicloxacillin |
|  | Other antibiotics | amikacin, chloramphenicol, clindamycin, doxycycline, gentamicin, metronidazole, nitrofurantoin, spectinomycin (EML only), sulfamethoxazole+trimethoprim | Tobramycin  Metenamine |
| **Watch** | Quinolones and fluoroquinolones | e.g. Ciprofloxacin, levofloxacin, moxifloxacin, norfloxacin |  |
|  |  |  | 2^nd^ gen cephalosporins: Cefuroxime |
|  | 3^rd^ generation cephalosporins (with or without beta-lactamase inhibitor) | e.g. Cefixime, ceftriaxone, cefotaxime, ceftazidime |  |
|  | Macrolides | e.g. Azithromycin, clarithromycin, erythromycin |  |
|  | Glycopeptides | e.g. teicoplanin, vancomycin |  |
|  | Antipseudomonal penicilins + beta-lactamase inhibitor | e.g. piperacillin - tazobactam |  |
|  | Carbapenems | e.g. meropenem, imipenem + cilastatin |  |
|  | Penems | e.g. faropenem |  |
| **Reserve** | Aztreonam |  |  |
|  | 4th generation cephalosporins | e.g. cefepime |  |
|  | 5^th^ generation cephalosporins | e.g. ceftaroline |  |
|  | Polymyxins | e.g. polymyxin B, colistin |  |
|  | Oxazolidinones | e.g. linezolid |  |
|  | Fosfomycin (IV) |  |  |
|  | Tigecyclin |  |  |
|  | Daptomycin |  |  |

* Antibiotics included in the access category only

**Table 3: Evaluation of antimicrobial spectrum and categorization of change**

| **ESCALATION** | **Added spectrum in new regime** |
| --- | --- |
| Cipro →Ampi/genta | Gram positives (Enterococci) |
| Erytro→ Ceftriaxon | Gram negatives |
| Cefotax→ Pip-taz | Anaerobes, enterococci |
| Ampi/genta→ Pip-taz | Anaerobes |
| Pc iv →TXS | Gram neg |
| Ampi→Doxy | Atypicals (Intracellular) |
| Pc→Clinda | Anaerobes and staphylococci |
| Genta→TXS | Streptococci |
| Cefotax→ Cipro/Metron | Anaerobes |
| Cefurox→Cefotax | Gram negatives |
| Pip-taz→Meropenems | ESBLs |
| Pc/Azitro→Cefotax | Gram negatives |
| Cefotaxime/Metron→Merop | ESBLs |
| Kloxa→Pc/Clinda | Anaerobes |
| Cefurox→ Pip-taz | Anaerobes, enterococci |
| Fenoxypc→Claritromycin | Atypicals (Intracellular) |
| Kloxa→Pc/Metro | Anaerobes |
| Cefotax/Metron→Meropenems | ESBLs |
| Pc/metro→Pc/genta | Gram negatives |
| Cefotaxime/Ampi→PipTazo | Anarobes |
| Pc→Doxy | Atypicals (Intracellular) |
| Cefurox→ TXS | Gram negatives |
| Cipro/genta→ TXS | Gram positives |
| Vanco/Clinda→ TXS | Gram negatives |
|  |  |
| **DE- ESCALATION** | **Reduced spectrum in new regime** |
| Pc/genta→Ampi | Gram negatives |
| Ampi/genta→ Cefuroxime | Enterococci, some gram negatives |
| Pip-taz→ Fenoxypc+cipro | Anaerobes |
| Cefotax→ Cipro | Gram positives |
| Clinda →Dicloxa | Anaerobes and ↓streptocci |
| Cefotaxime/Metron→Cipro/TXS | Anaerobes |
| Pip-taz→Cipro | Anaerobes and gram positives |
| Pc/genta→ Fenoxypc | Gram negatives, S aureus |
| Pc/genta→Amoxi | S aureus, Several gram negs |
| Cefurox→Cipro | Gram positives |
| TXS→Mecillinam | Gram positives, Several gram negs |
| Kloxa/Genta/Clinda→Pc | Gram negatives, S aureus, Anaerobes |
| Pc/clinda→Kloxa | Anaerobes |
| Cefurox/metro→Cefotax | Anaerobes |
| Pc/genta/metro→Ampi/genta | Anaerobes |
| Pc/genta→Pc/metro | Gram negatives |
| Kloxa /Clinda→Pc | S aureus, some anaerobes |
| Cefurox→Cipro | Gram positives |
| Ampi/genta→Amoxi | S aureus, Several gram negs |
| PipTaz→Cipro/Azitro | Anaerobes, enterococci |
| Ampi/genta→TXS | Enterococci |
| Kloxa/genta→Cipro | Gram positives |
| Pc/Kloxa→Fenoxypc | S aureus |
| Pc/genta→Fenoxypc | Gram neg, S aureus |
| Pc/genta/metro→Cipro/metro | Gram positives |
| Amoxi/Pivmecill→Cipro | Gram positives, enterococci |
| Ampi/genta→Cipro | Gram positives, incl enterococci |
|  |  |
| **UNCHANGED** |  |
| Cefotax↔ Pc/genta |  |
| Cefotax↔TXS |  |
| Pc/Cipro ↔ Pc/genta |  |
| Ampi/genta↔ Ceftriaxon/Pc |  |
| Pc/Cefotax↔Cefotax |  |
| Pc/Clinda/genta↔ Clinda/genta |  |
| Ampi/genta/Cefurox↔ Ampi/genta |  |
| Cefotax /genta↔Cefotax |  |
| Pip-Taz/Clinda↔Pip Taz | Increased penetration, but not broader antimicrobial spectrum |
